# Medical expenses and care pathways of patients with Pompe receiving myozyme: an observational study based on the French national healthcare database

**DOI:** 10.1186/s13023-025-03866-2

**Published:** 2025-10-31

**Authors:** Alicia Le Bras, Pascale De Lonlay, Shahram Attarian, Anaïs Brassier, Isabelle Durand-Zaleski, Pascal Laforêt, Pompe Study Group, Shahram Attarian, Pascal Laforêt, Azzeddine Arrassi, Anne-Laure Bedat-Millet, Anthony Béhin, Stéphane Beltran, Françoise Bouhour Fatima Bouibede, Catherine Caillaud, Brigitte Chabrol, Murielle Champeaux, Pascal Cintas, Jean-Baptiste Davion, Eva Diab, Joelle Deibener-Kaminsky, Florence Demurger, Caroline Espil, Florence Esselin, François Feillet, Maxime Fournier, Roseline Froissart, Karima Ghorab, Charlene Gillet, Magali Gorce, Dalil Hamroun, Jean-Yves Hogrel, Frédéric Huet, Elsa Krim, François Labarthe, Emmeline Lagrange, Françoise Lebeau, Claire Lefeuvre, Armelle Magot, Laurent Magy, Marcel Maillet-Vioud, Marion Masingue, Karine Mention, Maud Michaud, Aleksandra Nadaj-Pakleza, Sylvain Nollet, Adeline Not, Jean-Baptiste Noury, David Orlikowski, Caroline Paricio, Thierry Perez, Samia Pichard, Hélène Prigent, Dimitri Renard, Juliette Ropars, Sabrina Sacconi, Emmanuelle Salort-Campana, Guilhem Sole, Marco Spinazzi, Frédéric Taithe, Nadjib Taouagh, Céline Tard, Marine Tardieu, Ségolène Toquet, Guy Touati, Patrick Vandeputte, Annie Verschueren, Didier Vincent, Ulrike Walther-Louvier, Taissir Ziri

**Affiliations:** 1 APHP, DRCI-URC Eco Ile-de-France, Unité de Recherche Clinique en économie de la santé, 1 Place du Parvis Notre Dame, 75004 Paris, France; 2 APHP, Hôpital Necker-Enfants malades, Centre de référence des maladies héréditaires du métabolisme, Filière G2M, Imagine, INEM, Université Paris Cité, Paris, France; 3 APHM, Hôpital de la Timone, Centre de référence des Maladies Neuromusculaires et de la SLA, ERN-NMD, Marseille, France; 4 APHP, Hôpital Necker-Enfants malades, Centre de référence des maladies héréditaires du lysosome, Filière G2M, Imagine, Université Paris Cité, Paris, France; 5 APHP, Henri Mondor-Albert-Chenevier, Service de Santé Publique, Créteil, France; 6 CRESS, INSERM, INRA, Université de Paris, Paris, France; 7 APHP, Hôpital Raymond Poincaré, Neurology Department Garches, Nord-Est-Ile-de-France Neuromuscular Reference Center, Garches, France

**Keywords:** Glycogen storage disease type II, Enzyme replacement therapy, Cohort studies, Care pathways, Claims, Expenditures, Medical spending

## Abstract

**Background:**

Pompe disease is a rare, severe genetic multisystem disorder. It was one of the first genetic muscle diseases to benefit from an innovative therapy—enzyme replacement therapy (ERT)—more than 15 years ago. Despite this progress, data on patient management in community healthcare settings remain scarce, yet essential to understanding the dual economic and clinical burden of the disease. In this context, the French national healthcare claims database offers a valuable resource for comprehensive data collection. To our knowledge, this study is the first to provide a detailed, patient-level analysis of healthcare resource utilization for patients with Pompe disease. Our objective was to estimate the number of patients receiving ERT with alglucosidase alfa (Myozyme®), describe their care pathways, and assess associated medical expenses in both community settings and healthcare facilities across France.

**Method:**

Cases of Pompe disease were identified using the French national healthcare claims database aims for expensive drugs. All patients with at least one hospitalization for ERT with Myozyme® in France in 2022 were included. Healthcare resource utilization in 2022 was documented, and costs were assessed from the perspective of public health insurance. Costs drivers were analyzed using a generalised linear model. A supplementary analysis using 2023 data was also conducted.

**Results:**

A total of 154 patients were analyzed, including 24 with classic infantile-onset Pompe disease (IOPD) and 130 with late-onset Pompe disease (LOPD), with a mean age of 48.0 years. The annual healthcare cost per patient was €232,117 for IOPD patients and €358,768 for LOPD patients, nearly 96% of the costs were incurred inside hospitals. In addition to the unit cost of Myozyme® and related hospitalization, ventilator dependence and female sex emerged as major cost drivers. The 2023 data confirmed the robustness of the main findings, showing no substantial year-specific variations.

**Conclusion:**

This study provides robust real-world data to assess both hospital and non-hospital costs associated with Pompe disease in patients treated with Myozyme®. With an average of 81.9 medical or paramedical consultations per year and 49 days of hospitalization in 2022, the clinical burden imposed by Pompe disease is substantial, yet essential for patient care.

**Supplementary Information:**

The online version contains supplementary material available at 10.1186/s13023-025-03866-2.

## Introduction

Pompe disease (also known as glycogen storage disease type II, GSDII) is a rare and severe autosomal recessive disorder caused by a deficiency in the lysosomal enzyme acid α-glucosidase (GAA). This enzyme is essential for breaking down glycogen, and its deficiency leads to glycogen accumulation within lysosomes, especially in skeletal and cardiac muscle cells.

The clinical manifestations of Pompe disease vary widely [1, 2]. The infantile-onset form typically presents in the first few months of life with generalized hypotonia, muscle weakness, hypertrophic cardiomyopathy, and respiratory insufficiency. Without treatment, most affected infants die within the first year due to cardiorespiratory failure [3]. The late-onset form, in contrast, manifests as gradual muscle weakness, particularly in the proximal and axial muscles, often leading to respiratory insufficiency and motor decline over several decades, with respiratory failure being the leading cause of death [4].

Since 2006, enzyme replacement therapy (ERT) with recombinant human GAA (alglucosidase alfa, Myozyme®) has been the only approved treatment for Pompe disease in Europe and the United States. ERT significantly improved survival rates, with a 100% survival rate at 24 months [5], compared to a 9% survival rate in untreated infants [6]. However, long-term survivors, particularly those with late-onset Pompe disease (LOPD), initially experience significant benefits, such as stabilization of motor and respiratory functions, but often face a secondary decline in these functions after 3–5 years of treatment [7, 8]. ERT is also costly, with the list price of Myozyme® being €475 per 50 ml [9]. Patients require regular infusions, typically every two weeks, lasting about 4 h [10].

A systematic review of the health economics literature on Pompe disease identified only eight economic evaluations, despite a comprehensive literature search [11]. The review highlighted that most studies were of relatively poor quality. One real-world study in England [12] estimated the hospital economic burden (excluding ERT) at nearly $13,269 per year for children and $4,280 for adults [11]. Other studies [13, 14] estimated the annual cost of ERT (drug acquisition cost only) ranging from $503,118 in Canada to $547,835 in the United States for adult patients [11] per year. Cost-of-illness studies help estimate healthcare resource needs within the context of budget constraints, as healthcare costs are influenced by reimbursement policies.

Based on claims data from the French National Health Data System, this article aims to describe the resources consumed both in hospital and outpatient settings, as well as the associated costs, for patients with Pompe disease treated with Myozyme® in France. This study is, to our knowledge, the first to provide a patient-level description of healthcare resource use in both hospital and non-hospital settings.

## Methods

### Study design and data sources

This retrospective study on individual-level medical expenditures utilized data from the national healthcare claims database. Primarily used for billing purposes, this database includes demographic information and details on healthcare resource utilization both in the community (non-hospital settings) and across all hospitals and healthcare facilities in France [15]. It covers the entire population over their lifetime, with complete data linkage. The database records the type and date of procedures performed by healthcare professionals at patients' homes, in private clinics, or in health, medical, and social welfare centers. All outpatient medical and surgical procedures, imaging, reimbursable medications, home care services, and medical transport are coded. Hospital admissions are classified according to a diagnosis-related group (DRG) system.

### Study population

Myozyme® is exclusively used to treat patients with Pompe disease. The study population included all patients born before January 1, 2022, who received at least one infusion of Myozyme® in France during 2022. Patients under the age of 15 as of January 1, 2022, were classified as having classic infantile-onset Pompe disease (IOPD), while those aged 15 and older were classified as having late-onset Pompe disease (LOPD).

### Outcomes

Results were analyzed for the overall population and stratified by the two main disease forms (IOPD: under 15 years; LOPD: 15 years and older).

Socio-demographic characteristics, including age at first hospitalization in 2022, gender, and social deprivation index, were collected. Social deprivation index is based on sociodemographic factors [16] and the status of universal complementary health insurance (CMUc) delivered to people or families with an annual income less than the poverty threshold.

Associated health conditions were identified based on prior specific treatments, primary hospital admission diagnoses, or algorithms provided by the National Health Insurance [17].

Healthcare resource use and associated costs for Pompe disease patients were evaluated using 2022 data. Ambulatory care included consultations with general practitioners and specialists, paramedical services (e.g., nursing, physiotherapy, speech therapy), medications, laboratory tests, and some direct non-medical costs (e.g., transportation services, medical devices like wheelchair rentals or purchases). Hospital care expenditures encompassed inpatient care costs (including high-cost drugs and medical devices), rehabilitation, psychiatry, and home hospitalization. All expenditures were calculated based on reimbursements by the French social security system and expressed in Euros for 2022.

### Statistical methods

Descriptive analyses were conducted. Continuous variables were reported as means with standard deviations (± SD) and medians with interquartile ranges (Q1-Q3), while categorical variables were presented as frequencies and percentages.

A generalized linear model with a gamma distribution and a log link function was employed to examine the relationship between various explanatory variables and reimbursements by the French social security system, excluding all hospital stays associated with Myozyme® injections, which served as the dependent variable. The explanatory variables included age category (< 15 years, 15–60 years, ≥ 60 years), sex, social deprivation index, comorbidities (chronic cardiovascular and neurovascular diseases, chronic respiratory disease, psychiatric illness, cancer), use of antihypertensive treatment, ventilator dependence and wheelchair dependence. Covariate selection for the model was conducted by first assessing correlations among the aforementioned variables. Subsequently, variables were retained based on their statistical significance in bivariate analyses, with a p-value threshold of < 0.20. In the final model, p-values < 0.05 were considered indicative of statistical significance.

A supplementary analysis focusing on data from the year 2023 was conducted to assess the robustness of our findings. Given the anticipated decrease in the number of patients receiving Myozyme® during 2023—attributable to the introduction of Nexviadyme®, a therapy granted temporary market access in France —we elected to retain 2022 as the primary year for analysis to maximize patient inclusion. The year 2021 was excluded from the analysis due to its proximity to the peak of the COVID-19 pandemic, during which patients with Pompe disease experienced significant disruptions in their management.

All descriptive and statistical analyses were performed using R software, version 4.0.1 (The R Foundation) [18].

## Results

Between January 1 and December 31, 2022, 155 patients received Myozyme® in France. One patient was excluded from the analysis due to a date of birth after January 1, 2022.

Of the remaining 154 patients included in the analysis, 24 were classified as having IOPD (age under 15 years), and 130 were classified as having LOPD (age 16 years or older).

### Patient characteristics

The characteristics of patients receiving Myozyme® in 2022 are summarized in Table 1. The mean age of the cohort at inclusion was 48.0 years, and 53.9% of the patients were female.

**Table 1:** Characteristics and Health Status of Patients with Pompe Disease

Characteritic	IOPD (N = 24)	LOPD (N = 130)	ALL (N = 154)
Death in 2022—N(%)	1 (4.2%)	4 (3.1%)	5 (3.2%)
Sociodemographic characteristics			
Age–mean (± sd)	7.4 (± 4.4)	55.5 (± 15.1)	48.0 (± 22.4)
med [Q1–Q3]	6.5 [3.5–10.2]	55.5 [44.7–68.0]	51.6 [39.4–65.2]
Female—N (%)	8 (33.3%)	75 (57.7%)	83 (53.9%)
Social deprivation index—N (%)			
1st quantile (least deprived)	7 (29.2%)	30 (23.1%)	37 (24.0%)
2nd quantile	3 (12.5%)	20 (15.4%)	23 (14.9%)
3rd quantile	2 (8.3%)	20 (15.4%)	22 (14.3%)
4th quantile	3 (12.5%)	18 (13.8%)	21 (13.7%)
5th quantile (most deprived)	5 (20.8%)	34 (26.2%)	39 (25.3%)
Missing	4 (16.7%)	8 (6.1%)	12 (7.8%)
Comorbidities–N (%) includes five missing data points in LOPD			
Chronic cardioneurovascular diseases	6 (25.0%)	13 (10.4%)	19 (12.8%)
Chronic respiratory disease	7 (29.2%)	56 (44.8%)	63 (42.3%)
Psychiatric illness	0 (0.0%)	7 (5.6%)	7 (4.7%)
Cancer	0 (0.0%)	14 (11.2%)	14 (9.4%)
Treatment–N (%)			
Antihypertensives	1 (4.2%)	38 (30.4%)	39 (26.2%)
Dependency on medical devices at least one•–N (%)			
Ventilator dependence	8 (33.3%)	70 (53.8%)	78 (50.6%)
Wheelchair dependence	4 (16.7%)	30 (23.1%)	34 (22.1%)
Duration of Myozyme treatment in 2022 (in days) †–			
mean (± sd)	262.8 (± 123.3)	288.7 (± 110.8)	284.7 (± 112.8)
med [Q1-Q3]	332.5 [220.0–350.3]	350.0 [322.0–350.0]	350.0 [289.0–350.0]

†Period covering the admission date of the first hospital stay associated with Myozyme administration in 2022 to the discharge date of the last stay in the same year

•Reimbursement corresponds to the purchase or rental of a medical device in 2022

*IOPD* Infantile-Onset Pompe Disease; *LOPD* Late-Onset Pompe Disease; *Med* Median; *Q1-Q3* The interquartile range (IQR), representing the spread of the middle 50% of the data; *SD* Standard Deviation; *Q1* First Quartile; *Q3* Third Quartile

Among the 154 patients, reimbursement for the purchase or rental of a wheelchair was documented for 34 patients (22.1%). Additionally, 78 patients (50.6%) received reimbursement for the purchase or rental of an assisted ventilation device, including 8 patients with IOPD and 70 patients with LOPD.

### Characteristics of healthcare resources

Table 2 summarizes the primary healthcare resources utilized in 2022 by the 154 patients receiving Myozyme®. Further details on consultations with medical specialists are provided in Table S1. The average number of consultations (medical or paramedical) was 37.2 for IOPD patients and 89.9 for LOPD patients, resulting in an overall average of 81.9 for all patients.

**Table 2:** Healthcare use for the year 2022

Mean (± sd) Med [Q1-Q3]	IOPD (N = 24)	LOPD (N = 130)	ALL (N = 154)
General practitioner consultations	3.8 (± 4.0) 1.5 [1.0–7.2]	4.2 (± 4.0) 4.0 [1.0–6.0]	4.2 (± 4.0) 3.0 [1.0–6.0]
Medical specialists	3.0 (± 3.2) 2.0 [1.0–5.0]	5.9 (± 6.0) 4.5 [2.0–7.7]	5.5 (± 5.7) 4.0 [2.0–7.0]
Paramedical	30.4 (± 31.6) 17.0 [0.0–65.0]	79.8 (± 152.3) 52.5 [6.2–81.0]	72.2 (± 141.5) 45.5 [4.0–75.0]
• Nurses	2.3 (± 6.9) 0.0 [0.0–1.2]	30.8 (± 129.3) 1.0 [0.0–6.0]	26.4 (± 119.2) 1.0 [0.0–5.0]
• Physiotherapists	19.8 (± 25.7) 1.5 [0.0–35.5]	46.3 (± 50.9) 30.0 [0.0–72.5]	42.2 (± 48.8) 32 [0.0–68.7]
• Speech therapists	7.3 (± 18.7) 0.0 [0.0–0.0]	1.3 (± 8.2) 0.0 [0.0–0.0]	2.2 (± 10.7) 0.0 [0.0–0.0]
• Other paramedical professionals	1.0 (± 2.2) 0.0 [0.0–0.0]	1.4 (± 1.8) 0.0 [0.0–0.0]	1.4 (± 1.9) 0.0 [0.0–0.0]
Laboratory tests	1.3 (± 1.8) 1.0 [0.0–2.0]	4.0 (± 5.3) 2.0 [1.0–5.0]	3.6 (± 5.0) 2.0 [1.0–5.0]
Transportation	16.3 (± 18.8) 7.5 [0.0–34.0]	16.6 (± 14.0) 16.5 [2.0–26.0]	16.6 (± 14.8) 16.0 [2.0–26.7]
Number of days of hospitalization	62.7 (± 56.6) 50.5 [29.5–62.0]	46.5 (± 77.9) 26.0 [25.0–29.0]	49.0 (± 75.1) 27.0 [25.0–30.7]
• In a medical, surgical or obstetric department	37.3 (± 21.1) 29 [26–55]	40.4 (± 72.2) 26 [24–28]	40.0 (± 66.8) 26 [24–28]
• In follow-up care and rehabilitation department	2.4 (± 5.6) 0.0 [0.0–0.0]	3.2 (± 22.1) 0.0 [0.0–0.0]	3.0 (± 20.4) 0.0 [0.0–0.0]
• At home	23.0 (± 54.5) 0.0 [0.0–7.5]	2.9 (± 17.7) 0.0 [0.0–0.0]	6.0 (± 27.6) 0.0 [0.0–0.0]
Number of Myozyme administrations	27.1 (± 16.1) 25.0 [17.7–43.2]	20.1 (± 7.6) 24.0 [19.0–25.0]	21.2 (± 9.7) 24.0 [18.0–25.0]

*OPD* Infantile-Onset Pompe Disease; *LOPD* Late-Onset Pompe Disease; *Med* Median; *Q1-Q3* The interquartile range (IQR), representing the spread of the middle 50% of the data; *SD* Standard Deviation; *Q1* First Quartile; *Q3* Third Quartile

On average, patients with IOPD and LOPD spent 62.7 and 46.5 days in hospitals annually, respectively. Among IOPD patients, 4 (16.7%) had at least one hospitalization for rehabilitation care, and 6 (25.0%) experienced at least one home hospitalization. For LOPD patients, these figures were 19 (14.6%) and 9 (6.9%), respectively. With the exception of one patient, all individuals had been hospitalized at least once in a medical, surgical, or obstetric department, primarily for Myozyme® administration. The exceptional patient (LOPD) received all doses of Myozyme® through home hospitalization.

### Annual costs

From the perspective of the French social security system, the mean annual cost for patients with Pompe disease in 2022 was €232,117 (± €117,138) for IOPD patients, with 66.5% of this cost attributable to expensive medications (drug acquisition cost only) (see Table 3 and Table S2). For LOPD patients, the mean cost was €358,768 (± €154,733), with 81.5% allocated to expensive medications. The cost of Myozyme® accounted for over 92% of the total expenditure on expensive medications for these patients in 2022. Further details on hospital care costs can be found in Table S2.

**Table 3:** Annual Costs for Patients with Pompe Disease (2022)

Mean (± sd) Med [Q1-Q3]	IOPD (N = 24)	LOPD (N = 130)	ALL (N = 154)
General practitioner consultations	114 (± 119) 59 [17–200]	116 (± 115) 92 [25–164]	115 (± 115) 92 [25–171]
Medical specialists	178 (± 240) 147 [42–281]	432 (± 731) 299 [129–516]	393 (± 684) 278 [106–480]
Paramedical	770 (± 874) 481 [10–1,288]	1,614 (± 2,652) 975 [162–1,830]	1,483 (± 2,477) 931 [111–1,773]
• Nurses	38 (± 134) 0 [0–25]	387 (± 1,754) 9 [0–45]	333 (± 1,616) 7 [0–41]
• Physiotherapists	413 (± 577) 32 [0–713]	1 077 (± 1,313) 797 [0–1,644]	974 (± 1,249) 665 [0–1,517]
• Speech therapists	276 (± 698) 0 [0–0]	49 (± 321) 0 [0–0]	84 (± 409) 0 [0–0]
• Other paramedical professionals	43 (± 85) 0 [0–36]	101 (± 185) 31 [0–104]	92 (± 174) 30 [0–96]
Laboratory tests	52 (± 76) 27 [0–69]	145 (± 219) 70 [25–156]	130 (± 206) 67 [17–142]
Transportation	3,059 (± 4,558) 1,350 [0–5,210]	3,513 (± 4,036) 2,274 [461–5,329]	3,442 (± 3,957) 2,178 [284–5,329]
Medical devices	4 304 (± 9,393) 660 [0–3,813]	2,375 (± 2,563) 1,846 [0–3,479]	2,676 (± 4,392) 1,731 [0–3,529]
Drugs (dispensed in the community)	721 (± 1,656) 242 [121–606]	669 (± 990) 276 [127–834]	677 (± 1,113) 265 [127–797]
Sick-leave benefit	NA	4,881 (± 10,228) 0 [0–2,414]	4,120 (± 9,558) 0 [0–695]
Hospital care	222,767 (± 112,415) 185,151 [140,110–311,746]	344,895 (± 153,392) 335,698 [251,601–422,908]	325,863 (± 153,991) 323,125 [225,582–398,378]
• ERT with myozyme (drug only)	147,829 (± 97,240) 120,107 [87,584- 189,224]	268,888 (± 134,452) 290,255 [189,708–352,401]	250,022 (± 136,395) 255,906 [255,907–212,241]
Other	152 (± 384) 0 [0–94]	128 (± 496) 0 [0–0]	132 (± 479) 0 [0–0]
Total	232,117 (± 117,138) 191,438 [142,176–316,221]	358,768 (± 154,733) 345,329 [261,668–438,814]	339,031 (± 156,120) 335,022 [238,071–421,356]

*ERT* Enzyme Replacement Therapy; *IOPD* Infantile-Onset Pompe Disease; *LOPD* Late-Onset Pompe Disease; *Med* Median; *Q1-Q3* The interquartile range (IQR), representing the spread of the middle 50% of the data; *SD* Standard Deviation; *Q1* First Quartile; *Q3* Third Quartile

Two patients with IOPD and five patients with LOPD received Myozyme® treatment at home. The average cost per day of home hospitalization, excluding the cost of Myozyme®, was €399 and €342, respectively. In comparison, the cost of a day of hospitalization in a medical department was €372.

The average cost per administration of Myozyme® was €5,785 (± €2,424) for patients with IOPD and €13,525 (± €4,048) for patients with LOPD, resulting in an overall average of €12,319 (± €4,757) across all patients.

### General linear model

The general linear model (Table 4) found that sex, ventilator dependence and wheelchair dependence were significantly associated to reimboursment (excluding all hospital stays associated with Myozyme injection). Patients with ventilator dependence had a rate ratio of 2.32 (*p* < 0.05), indicating that their average reimbursements were approximately 2.32 times higher than those without ventilator dependence.

**Table 4:** Generalized Linear Model Results: Association Between Patient Characteristics and 2022 Healthcare Costs (Excluding Myozyme®-Related Hospitalizations)

Patients characteristics		Rate ratio	95% IC	*P*-value
Intercept		4,970	(3436–7458)	< 0.001
Age category	< 15 years	1.00	1.00	
	15–59 years	1.44	(0.94–2.14)	0.08
	≥ 60 years	0.92	(0.57–1.46)	0.74
Sex	Male	1.00	1.00	0.02
	Female	1.40	(1.03–1.87)	
Ventilator dependence	No	1.00	1.00	< 0.001
	Yes	2.32	(1.73–3.12)	
Wheelchair dependence	No	1.00	1.00	0.06
	Yes	1.40	(1.0–2.00)	

### Supplementary analysis

A supplementary analysis was conducted on data from January 1 to December 31, 2023, encompassing 136 patients who received Myozyme® in France. Of these, 14 were classified as having IOPD, and 122 were classified as having LOPD. The results of this analysis are presented in the supplementary files (Tables S3, S4 and S5). Comparative analysis of the 2022 and 2023 data confirmed the robustness of our findings, with a variation in the average management cost per patient of 3.97% for IOPD (€232,117 in 2022 vs. €223,247 in 2023) and 0.66% for LOPD (€358,768 in 2022 vs. €356,412 in 2023) between the two years.

## Discussion

This retrospective analysis of 154 patients identified from the French national healthcare claims database of patients with Pompe disease, who received at least one Myozyme® treatment in 2022, found that patients with IOPD spent an average of 62.7 days per year in the hospital and had an average of 37.2 medical or paramedical consultations. For patients with LOPD, these figures were 46.5 days and 89.9 consultations, respectively. The high number of consultations is partly due to the essential care provided by paramedical professionals in community settings.

The mean annual cost of healthcare and paid sick leave for patients with Pompe disease was estimated to be €232,117 (± €117,138) for IOPD patients and €358,768 (± €154,733) for LOPD patients. To assess the robustness of our findings, we conducted a sensitivity analysis using 2023 data from the French national healthcare database. Results were consistent with those obtained for 2022, suggesting that the economic burden estimates are stable over time and not influenced by year-specific variations. The primary cost driver was Myozyme®, accounting for nearly 75% of the annual expenses. After excluding costs directly related to Myozyme infusions and associated hospital stays, two additional major cost drivers were identified: ventilator dependence and female sex. Higher costs among ventilator-dependent patients were expected due to the need for specialized equipment, intensive monitoring, and greater healthcare resource utilization. Female sex was also associated with higher costs. This may reflect differences in body weight (affecting non-drug-related care), disease severity, or healthcare-seeking behaviors. Further research is warranted to better understand this association.

Our findings are consistent with published data from the French Pompe Disease Registry [19], which reported that 22% of patients with LOPD require a wheelchair (compared to 23.1% in our study), and 60% require respiratory support equipment (compared to 53.8% in our study). However, dependency rates in our study may be underestimated, as only patients who received at least one reimbursement in 2022 for the purchase or rental of an assisted ventilation device or a wheelchair were included. Furthermore, some patients may have acquired a wheelchair or respiratory support equipment in previous years and were therefore not captured in the database.

Although current ERT offers numerous benefits, it does have limitations, particularly concerning immune tolerance. Nevertheless, it is clear that patients have experienced prolonged survival and improved clinical outcomes. However, the high cost of these therapies limits their availability and prescription in many countries.

For indicative comparison only, monetary values were standardized as follows: euro values from 2022 were converted to 2022 USD using the average 2022 exchange rate (1 EUR ≈ 1.0530 USD) [20]; euro values from 2013 were converted to 2013 USD using the average 2013 exchange rate (1 EUR ≈ 1.3281 USD) [20]; and U.S. dollar values from 2017 were adjusted to 2022 USD using the U.S. Consumer Price Index for “Prescription Drugs” (CPI 2017 = 520, CPI 2022 = 534; inflation adjustment factor ≈1.0269) [21]. These conversions are provided for indicative comparison purposes only and do not reflect actual drug pricing mechanisms across countries. Our results for IOPD (€120,107, approximatively $126,473 in 2022 USD [20]) was consistent with the findings of the systematic literature review by Schoser et al. [11], which reported drug acquisition costs ranging from $37,132 to $207,604 in 2017. Adjusted for inflation, these correspond to $38,131 to $213,189 in 2022 USD (inflation adjustment factor = 1.0269). However, our cost estimate for LOPD (€290,255 or $305,639 in 2022 USD) was lower than the estimates reported in the same review [11]: $503,118 in Canada [13] and $547,835 in the United States [14] (2017 values adjusted to $516,652 and $562,572 respectively in 2022 USD using the same inflation adjustment factor). A possible explanation for this cost difference is the significant price reduction of Myozyme® in France—from €525 per 50 mg in 2013 ($697 in 2013 using the 2013 exchange rate) to €475 per 50 mg in 2022 [9] ($500 using the 2022 exchange rate). In contrast, the drug remained more expensive in North America, with prices reaching approximately $1,000 per 50 mg in the U.S. (2012) [14] and $840 per 50 mg in Canada (2014) [13]. Another contributing factor may be the variation in cost estimates based on patient weight, as Myozyme® dosing is weight-dependent.

The strength of our study lies in the use of a national, comprehensive database. At the individual level, this database includes all health services, whether reimbursed or not, across both community settings and all hospitals and healthcare facilities in France.

We identified the following limitations. First, we assumed that all patients aged 15 years and older had the late-onset Pompe disease (LOPD) form. While this is likely accurate for the majority of patients, as 88% of LOPD patients are 40 years or older. Second, our analysis focused only on patients who received ERT with Myozyme® in 2022. The identification of Pompe disease patients in the French national hospital discharge database is only possible when expensive drugs, such as Myozyme®, are administered. According to the French Pompe disease registry, 224 were living with Pompe disease in 2022. Our analysis focuses on the healthcare utilization of 69% of all patients with Pompe disease in France that year.

No comparison could have been made between patients treated with Myozyme® and those untreated because the populations are not comparable. The primary reason for the absence of treatment is the asymptomatic presentation of the disease. The second reason is patient refusal, often due to hesitation about biweekly hospital visits and concerns about potential side effects. In the absence of feasible randomized clinical trials, our study provides valuable insights into the real-world experiences of patients, encompassing aspects such as management, disease impact, and treatment effects on daily life. Recently, the integration of the French National Rare Diseases Registry (BNDMR) with the French National Healthcare database has become possible. This linkage is anticipated to enhance our study by enabling the identification and inclusion of patients not treated with Myozyme®, who were previously unidentifiable within the SNDS alone.

This study highlights several considerations relevant to public health policy concerning the management of Pompe disease. ERT with Myozyme® involves an annual cost estimated at approximately €255,00 per patient in France. Considering that Pompe disease is a severe condition, this expenditure raises concerns about long-term financial sustainability and underscores the need to assess the cost-effectiveness of such therapies, particularly in the context of limited resources. Without treatment, most affected infants do not survive beyond the first year of life.

ERT for Pompe disease requires biweekly hospital infusions, which can be burdensome for patients residing far from treatment centers. A study has demonstrated the feasibility and safety of home-based ERT with Myozyme [22]. However, home-based ERT with Myozyme® is rare in France. Hospital-based care not only ensures the safe administration of ERT but also provides patients with opportunities to discuss their treatment and health concerns directly with their healthcare providers. While home-based ERT may offer benefits in terms of convenience and reduced hospital resource utilization, we have to consider the individual needs and circumstances of each patient when determining the most appropriate treatment setting.

## Conclusion

In conclusion, the analysis of the French national healthcare claims database provides additional insights into the Pompe disease patient registry by describing healthcare resource utilization (both in community settings and across all hospitals and healthcare facilities, including paid sick leave) and estimating the associated costs. In addition to the unit cost of Myozyme® and related hospitalization, ventilator dependence and female sex emerged as major cost drivers. With an average of 81.9 medical or paramedical consultations per year and 49.0 days of hospitalization in 2022, the clinical burden of Pompe disease is substantial. Analysis of 2023 data reaffirmed the robustness of the results, with consistent findings across both years.

## Supplementary Information

Additional file 1.

## Data Availability

The data that support the findings of this study are available from the corresponding author upon reasonable request.
