# Supplementary material for: Medical expenses and care pathways of patients with Pompe receiving myozyme: an observational study based on the French national healthcare database
Source: Orphanet J Rare Dis. 2025 Oct 31;20:554. doi: 10.1186/s13023-025-03866-2 (PMC12577294; doi:10.1186/s13023-025-03866-2)
Supplement: Supplementary file 1 — Additional file 1. [file 13023_2025_3866_MOESM1_ESM.docx]

**Table S1.** Consultations with health professionals for patients with Pompe disease receiving myozyme in 2022

| Mean (±sd)  med [Q1-Q3] | **IOPD**  **(N = 24)** | **LOPD**  **(N = 130)** | **ALL**  **(N =154)** |
| --- | --- | --- | --- |
| **Medical specialists** | **3.0 (3.2)**  **2.0 [1.0-5.0]** | **5.9 (6.0)**  **4.50 [2.0-7.7]** | **5.5 (5.7)**  **4.0 [2.0-7.0]** |
| *Psychiatrist* | 0.0 (0.0)  0.0 [0.0-0.0] | 0.6 (3.0)  0.0 [0.0-0.0] | 0.51 (2.74)  0.0 [0.0-0.0] |
| *Radiologist* | 0.1 (0.3)  0.0 [0.0-0.0] | 1.2 (1.6)  1.0 [0.0-2.0] | 1.06 (1.6)  0.0 [0.0-1.0] |
| *Cardiologist* | 0.3 (0.5)  0.0 [0.0-0.2] | 0.7 (1.17)  0.0 [0.0-1.0] | 0.6 (1.1)  0.0 [0.0-1.0] |
| *Surgeon* | 0.8 (1.4)  0.0 [0.0-1.0] | 0.2 (0.7)  0.0 [0.0-0.0] | 0.3 (0.8)  0.0 [0.0-0.0] |
| *Anesthesiologist* | 0.2 (0.6)  0.0 [0.0-0.0] | 0.1 (0.3)  0.0 [0.0-0.0] | 0.1 (0.4)  0.0 [0.0-0.0] |
| *Neurologist* | 0.2 (0.4)  0.0 [0.0-0.0] | 0.3 (0.6)  0.0 [0.0-0.0] | 0.3 (0.6)  0.0 [0.0-0.0] |
| *Pulmonologist* | 0.0 (0.2)  0.0 [0.0-0.0] | 0.4 (0.67)  0.0 [0.0-1.0] | 0.32 (0.64)  0.0 [0.0-0.0] |
| *Gastroenterologist* | 0.0 (0.0)  0.0 [0.0-0.0] | 0.23 (0.58)  0.0 [0.0-0.0] | 0.19 (0.54)  0.0 [0.0-0.0] |
| *Internist* | 0.04 (0.20)  0.0 [0.0-0.0] | 0.18 (0.43)  0.0 [0.0-0.0] | 0.16 (0.40)  0.0 [0.0-0.0] |
| *Other* | 1.29 (1.65)  1.0 [1.0-2.0] | 2.0 (3.6)  1.0 [0.0-3.0] | 1.9 (3.4)  1.0 [0.0-2.0] |

Abbreviations: IOPD – Infantile-Onset Pompe Disease; LOPD – Late-Onset Pompe Disease; Med - Median; Q1-Q3 - The interquartile range (IQR), representing the spread of the middle 50% of the data; SD – Standard Deviation; Q1 – First Quartile; Q3 – Third Quartile.

**Table S2.** Hospitalization costs for patients with Pompe disease receiving myozyme in 2022

| Mean (±sd)  med [Q1-Q3] | **IOPD**  **(N = 24)** | **LOPD**  **(N = 130)** | **ALL**  **(N =154)** |
| --- | --- | --- | --- |
| **Hospitalization in a medical, surgical or obstetric department** | 180,965 (±116,503)  149,381 [102,375-236,450] | 335,570 (±164,621)  334,224 [242,937-422,908] | 311,476 (±167,495)  312,884 [204,584 – 393,659] |
| - *Expensive drugs* | *121,868 (*±*84,805)*  *105,393 [72,905-163,455]* | *284,736 (±167,181)*  *290,740 [187,527-361,007]* | *259,354 (±167,806)*  *250,279 [124,715-347,434]* |
| **Hospitalization in a follow-up care and rehabilitation department** | 1,517  (±4,107)  0 [0-0] | 1,030  (±6,503)  0 [0-0] | 1,106  (±6,183)  0 [0-0] |
| **Hospitalization at home** | 40,285 (±97,880)  0 [0-4,961] | 8,295  (±44,976)  0 [0-0] | 13,281  (±57,282)  0 [0-0] |
| - *Expensive drugs* | *32,466 (*±*93,408)*  0 [0-0] | *7,622 (±43,556)*  0 [0-0] | *11,494 (±54,707)*  0 [0-0] |
| **TOTAL – Hospital care** | 222,767 (±112,415)  185,151 [140,110-311,746] | 344,895 (±153,392)  335,698 [251,601-422,908] | 325,863 (±153,991)  323125 [225,582-398,378] |

Abbreviations: IOPD – Infantile-Onset Pompe Disease; LOPD – Late-Onset Pompe Disease; Med - Median; Q1-Q3 - The interquartile range (IQR), representing the spread of the middle 50% of the data; SD – Standard Deviation; Q1 – First Quartile; Q3 – Third Quartile.

**Table S3.** Characteristics and Health Status of Patients with Pompe Disease in 2023

| **Characteritic** | **IOPD**  **(N = 14)** | **LOPD**  **(N = 122)** | **ALL**  **(N = 136)** |
| --- | --- | --- | --- |
| **Death in 2023 – N(%)** | 0 (0.0%) | 4 (3.3%) | 4 (2.9%) |
| **Sociodemographic characteristics** |  |  |  |
| Age – mean (±sd)  med [Q1-Q3] | 6.5 (±4.1)  6.5 [4.0-9.7] | 53.8 (±15.4)  54.2 [44.7-64.7] | 48.9 (±20.6)  52.2 [40.5 – 62.5] |
| Female – N (%) | 6 (42.9%) | 68 (55.7%) | 74 (54.4%) |
| **Social deprivation index -** N (%) |  |  |  |
| *1st quantile* (least deprived) | 4 (28.6%) | 31 (25.4%) | 35 (25.7%) |
| *2nd quantile* | 2 (14.3%) | 22 (18.0%) | 24 (17.7%) |
| *3rd quantile* | 2 (14.3%) | 23 (18.9%) | 25 (18.4%) |
| *4th quantile* | 0 (0.00%) | 14 (11.5%) | 14 (10.3%) |
| *5th quantile* (most deprived) | 5 (35.7%) | 22 (18.0%) | 27 (19.8%) |
| *Missing* | 1 (7.1%) | 10 (8.2%) | 11 (8.1%) |
| **Comorbidities** – N (%)  *includes five missing data points in LOPD* |  |  |  |
| Chronic cardioneurovascular diseases | 5 (35.7%) | 8 (6.7%) | 13 (9.7%) |
| Chronic respiratory disease | 3 (21.4%) | 49 (40.9%) | 52 (38.8%) |
| **Psychiatric illness** | 0 (0.0%) | 5 (4.2%) | 5 (3.7%) |
| Cancer | 0 (0.0%) | 11 (9.2%) | 11 (8.2%) |
| **Treatment –** N (%) |  |  |  |
| **Antihypertensives** | 2 (14.3%) | 34 (28.3%) | 36 (26.9%) |
| Dependency**on**medical**devices at least one** • – N (%) |  |  |  |
| Ventilator dependence | 3 (21.4%) | 60 (49.2%) | 63 (46.3%) |
| Wheelchair dependence | 2 (14.3%) | 19 (15.6%) | 21 (15.4%) |
| **Duration of Myozyme treatment in 2023 (in days)** †–  mean (±sd)  med [Q1-Q3] | 259.6 (132.0)  339.0 [147.2-354.2] | 279 (118.3)  349.0 [266.0-350.0] | 277 (119.4)  347.0 [251.5-350.0] |

† Period covering the admission date of the first hospital stay associated with Myozyme administration in 2023 to the discharge date of the last stay in the same year.

• Reimbursement corresponds to the purchase or rental of a medical device in 2023.

Abbreviations: IOPD – Infantile-Onset Pompe Disease; LOPD – Late-Onset Pompe Disease; Med - Median; Q1-Q3 - The interquartile range (IQR), representing the spread of the middle 50% of the data; SD – Standard Deviation; Q1 – First Quartile; Q3 – Third Quartile.

**Table S4.** Healthcare use for the year 2023

| Mean (±sd)  med [Q1-Q3] | **IOPD**  **(N = 14)** | **LOPD**  **(N = 122)** | **ALL**  **(N =136)** |
| --- | --- | --- | --- |
| **General practitioner consultations** | 3.4 (±4.8)  1.0 [1.0-3.7] | 4.7 (±4.6)  4.0 [2.0-6.0] | 4.6 (±4.6)  3.0 [1.0-6.0] |
| **Medical specialists** | 4.4 (±4.3)  3.0 [1.2-5.7] | 6.4 (±5.5)  5.0 [2.0-8.0] | 6.2 (±5.4)  5.0 [2.0-8.0] |
| **Paramedical** | 30.2 (±36.5)  15.5 [0.0-53.5] | 74.8 (±139.1)  49.0 [12.2-84.5] | 70.2 (±132.9)  48.0 [6.7-82.2] |
| - *Nurses* | 5.9 (±21.6)  0.0 [0.0-0.0] | 27.2 (±119.1)  1.0 [0.0-5.0] | 25.0 (±113.1)  1.0 [0.0-4.2] |
| - *Physiotherapists* | 17.1 (±22.0)  0.0 [0.0-34.0] | 45.2 (±48.3)  36.5 [0.0-77.0] | 42.3 (±47.1)  33.0 [0.0-71.0] |
| - *Speech therapists* | 6.2 (±16.1)  0.0 [0.0-0.0] | 0.9 (±5.7)  0.0 [0.0-0.0] | 1.5 (±7.6)  0.0 [0.0-0.0] |
| - *Other paramedical professionals* | 1.1(±2.6)  0.0 [0.0-0.0] | 1.5 (±2.4)  1.0 [0.0-0.0] | 1.5 (±2.4)  1.0 [0.0-0.0] |
| **Laboratory tests** | 0.1 (±0.3)  0.0 [0.0-0.0] | 3.0 (±3.7)  2.0 [0.2-4.0] | 2.7 (3.6)  1.5 [0.0-4.0] |
| **Transportation** | 22.4 (±28.1)  15.5 [0.7-29.0] | 15.4 (±14.0)  15.5 [1.0-25.7] | 16.2 (±16.0)  15.5 [1.0-26.0] |
| **Number of days of hospitalization** | 65.9 (±94.9)  43 [28.3-50.8] | 32.3 (±48.9)  26.0 [24.0-28.8] | 35.8 (±55.8)  26.0 [24.0-29.0] |
| - *in a medical, surgical or obstetric department* | *36.4 (*±*17.6)*  *31.5 [25.7-50.0]* | *23.9 (*±*8.1)*  *26.0 [23.0-28.0]* | *25.2 (*±*10.2)*  *26 [23-29]* |
| - *in follow-up care and rehabilitation department* | 1. *(*±*0.0)* 2. *0.0 [0.0-0.0]* | *1.20 (*±*8.0)*  *0.0 [0.0-0.0]* | *1.0 (*±*7.5)*  *0.0 [0.0-0.0]* |
| - *At home* | *29.5 (*±9*6.9)*  *0.0 [0.0-0.0]* | *7.4 (*±*47.8)*  *0.0 [0.0-0.0]* | *9.6 (*±*54.8)*  *0.0 [0.0-0.0]* |
| **Number of Myozyme administrations** | *26.6 (±15.4)*  *26.0 [18.5-35.7]* | *20.0 (±8.2)*  *24.0 [17.2-26.0]* | *20.7 (±9.4)*  *24.5 [17.0-26.0]* |

Abbreviations: IOPD – Infantile-Onset Pompe Disease; LOPD – Late-Onset Pompe Disease; Med - Median; Q1-Q3 - The interquartile range (IQR), representing the spread of the middle 50% of the data; SD – Standard Deviation; Q1 – First Quartile; Q3 – Third Quartile.

**Table S5.** Annual Costs for Patients with Pompe Disease (2023)

| Mean (±sd)  Med [Q1-Q3] | **IOPD**  **(N = 14)** | **LOPD**  **(N = 122)** | **ALL**  **(N =136)** |
| --- | --- | --- | --- |
| **General practitioner consultations** | 91 (±123)  25 [21-128] | 129 (±125)  100 [35-175] | 125 (±125)  97 [25-175] |
| **Medical specialists** | 269 (±325)  117 [87-295] | 397 (±357)  287 [162-514] | 384 (±355)  275 [145-504] |
| **Paramedical** | 710 (±810)  368 [0-1,171] | 1,560 (±2,481)  1,056 [259-1,938] | 1,472 (±2,376)  1,005 [206-1,912] |
| - *Nurses* | *67 (±247)*  *0 [0-0]* | *370 (±1,786)*  *5 [0-25]* | *339 (±1,506)*  *4 [0-24]* |
| - *Physiotherapists* | *374 (±471)*  *0 [0-784]* | *1 066 (±1,331)*  *698 [0-1760]* | *995 (±1,286)*  *669 [0-1,678]* |
| - *Speech therapists* | *224 (±580)*  *0 [0-0]* | *36 (±223)*  *0 [0-0]* | *55 (±409)*  *0 [0-0]* |
| - *Other paramedical professionals* | *45 (±128)*  *0 [0-0]* | *88 (±133)*  *40 [0-115]* | *83 (±133)*  *30 [0-107]* |
| **Laboratory tests** | 2 (±9)  0 [0-0] | 99 (±146)  52 [3-115] | 89 (±141)  44 [0-100] |
| **Transportation** | 3,353 (±3,402)  3,174 [79-5,714] | 3,123 (±3,858)  2,014 [228-4,452] | 3,147 (±3,802)  2,060 [219-4,664] |
| **Medical devices** | 2,861 (±5,189)  21 [0-3,738] | 2,312 (±3,771)  1,587 [0-3,232] | 2,369 (±3,920)  1,267 [0-3,232] |
| **Drugs (dispensed in the community)** | 339 (±386)  224 [115-449] | 889 (±2,139)  273 [112-711] | 833 (±2,035)  272 [110-662] |
| **Sick-leave benefit** | NA | 5,738 (±11,486)  0 [0-2,308] | 5,147 (±11,014)  0 [01,749] |
| **Hospital care** | 215,596 (±92,526)  206,232 [148,604- 269,798] | 342,084 (±177,971)  323,734 [248-958- 418,759] | 329,062 (±175,219)  301,489 [242,832-411,234] |
| - *ERT with myozyme (drug only)* | *128,097 (±85,228)*  *107,573 [66,520- 167,309]* | *269,648 (±148,722)*  *271,161 [179,533-378,352]* | *255,076 (±149,626)*  *264,089 [137,132-377,962]* |
| **Other** | 26 (60)  0 [0-0] | 81 (±272)  0 [0-0] | (±76 259)  0 [0-0] |
| **TOTAL** | **223,247 (±92,367)**  **219,163 [164,674-275,075]** | **356,412 (±178,441)**  **343,413 [261,132-440,603]** | **342,704 (±176,098)**  **318,701 [254,242-426,484]** |

**Abbreviations:** ERT – Enzyme Replacement Therapy; IOPD – Infantile-Onset Pompe Disease; LOPD – Late-Onset Pompe Disease; Med - Median; NA – Not applicable ; Q1-Q3 - The interquartile range (IQR), representing the spread of the middle 50% of the data; SD – Standard Deviation; Q1 – First Quartile; Q3 – Third Quartile.
